# EQ-5D-Y-5L: developing a revised EQ-5D-Y with increased response categories

**DOI:** 10.1007/s11136-019-02115-x

**Published:** 2019-02-09

**Authors:** Simone Kreimeier, Mimmi Åström, Kristina Burström, Ann-Charlotte Egmar, Narcis Gusi, Michael Herdman, Paul Kind, Miguel A. Perez-Sousa, Wolfgang Greiner

**Affiliations:** 1 Department of Health Economics and Health Care Management, School of Public Health, Bielefeld University, Universitätsstraße 25, 33615 Bielefeld, Germany; 2 Health Outcomes and Economic Evaluation Research Group, Stockholm Centre for Healthcare Ethics, Department of Learning, Informatics, Management and Ethics, Karolinska Institutet, 171 77 Stockholm, Sweden; 3 Equity and Health Policy Research Group, Department of Public Health Sciences, Karolinska Institutet, 171 77 Stockholm, Sweden; 4 Health Care Services, Stockholm County Council, 171 77 Stockholm, Sweden; 5 Department of Public Health and Medicine, Health-Promotion Interventions and Resilience, The Swedish Red Cross University College, 141 57 Huddinge, Sweden; 6 Faculty of Sport Sciences, University of Extremadura, Badajoz, Spain; 7 Office of Health Economics, London, UK; 8 Academic Unit of Health Economics, Leeds Institute of Health Sciences, University of Leeds, Leeds, UK; 9 Centre for Health Economics, Management and Policy, Higher School of Economics, St Petersburg, Russia

**Keywords:** EQ-5D-Y, EQ-5D-Y-3L, EQ-5D-Y-5L, Health-related quality of life (HRQoL), Children, Adolescent

## Abstract

**Purpose:**

EQ-5D-Y is a generic measure of health status for children and adolescents aged 8–15 years. Originally, it has three levels of severity in each dimension (3L). This study aimed to develop a descriptive system of EQ-5D-Y with an increased number of severity levels and to test comprehensibility and feasibility.

**Methods:**

The study was conducted in Germany, Spain, Sweden and the UK. In Phase 1, a review of existing instruments and focus group interviews were carried out to create a pool of possible labels for a modified severity classification. Participants aged 8–15 rated the severity of the identified labels in individual sorting and response scaling interviews. In Phase 2, preliminary 4L and 5L versions were constructed for further testing in cognitive interviews with healthy participants aged 8–15 years and children receiving treatment for a health condition.

**Results:**

In Phase 1, a total of 233 labels was generated, ranging from 37 (UK) to 79 labels (Germany). Out of these, 7 to 16 possible labels for each dimension in the different languages were rated in 255 sorting and response scaling interviews. Labels covered an appropriate range of severity on the health continuum in all countries. In Phase 2, the 5L version was generally preferred (by 68–88% of the participants per country) over the 4L version.

**Conclusions:**

This multinational study has provided a version of the EQ-5D-Y with 5 severity levels in each dimension. This extended version (EQ-5D-Y-5L) requires testing its psychometric properties and its performance compared to that of the original EQ-5D-Y-3L.

**Electronic supplementary material:**

The online version of this article (10.1007/s11136-019-02115-x) contains supplementary material, which is available to authorized users.

## Introduction

Since 2010, the EQ-5D-Y has been available as a ‘Youth’ version of the EQ-5D, for children and adolescents aged 8–15 years. The instrument was developed using the standard three-level (3L) format of the EQ-5D descriptive system (adult version). Like the EQ-5D, the current EQ-5D-Y-3L descriptive system comprises five dimensions of health; ‘mobility’, ‘looking after myself’, ‘doing usual activities’, ‘having pain or discomfort’, ‘feeling worried, sad or unhappy’. Each dimension has three levels of severity, resulting in a total of 243 possible health states. Although the same dimension and response option structure as used in the EQ-5D-3L was retained, the wording and layout were modified to be suitable for children and adolescents [1–3]. The EQ-5D-Y-3L has demonstrated its feasibility in children and adolescents with different health conditions [4–6].

In 2011, the EQ-5D-5L, a five-level (5L) version of the EQ-5D for adults, was introduced, with the aim of reducing the instrument’s ceiling effects and enhancing sensitivity, especially in milder health conditions [7]. Testing of the 5L adult version has shown that it works as well or better than the 3L in various conditions and settings [8–10].

As with the 3L adult version, there is evidence of ceiling effects for the EQ-5D-Y-3L and it has been criticized for being overly simplistic and potentially insensitive to small changes in health status [6, 11, 12]. In contrast to the EQ-5D-Y-3L, the majority of generic health-related quality of life (HRQoL) instruments for children and adolescents, such as the KINDL or PedQL [13, 14], use response options with more than three levels of severity. Expanding the number of severity levels in each dimension of the EQ-5D-Y might help to reduce ceiling effects and improve sensitivity.

The aim of the present study was to develop a descriptive system of the EQ-5D-Y with an increased number of severity levels and to test the comprehensibility and feasibility of the extended version. The number of levels in the final version was not defined a priori as a further aim of the study was to assess children’s opinion and the acceptability of using versions with 4 or 5 levels of severity in each dimension.

## Methods

This study was conducted in Germany, Spain, Sweden and the UK between May 2014 and June 2018. Ethical approval was obtained in each country. The study had two phases. In phase 1, potential severity labels were identified, sorting and response scaling interviews were conducted, and alternative 4L and 5L versions of EQ-5D-Y were developed for each country. In phase 2, both versions were tested for comprehensibility and feasibility and children’s opinion about the two versions were elicited. In both phases, a common standardized protocol was used to ensure that the same procedures were followed in each country.

### Phase 1

#### Identifying a pool of labels for the severity levels

##### Procedure

A pool of possible labels for the extension of EQ-5D-Y-3L severity levels was developed from a review of HRQoL instruments and by focus group interviews. Existing generic as well as disease-specific HRQoL instruments for children in the four different languages were included with the aim of identifying labels which covered the full range of severity. Dictionaries and thesauruses were used to search for synonyms of previously identified labels. When deciding which labels to include in the final pool, the lexical structure of the EQ-5D-Y was taken into account. Only labels describing the ‘quantity’ or ‘intensity’ of health problems (e.g., a lot of, slight) were included and, e.g., terms relating to frequencies, were excluded.

In addition, each country conducted two focus group interviews, one with children aged 8–10 and one with participants aged 11–15, as it was assumed that younger children would be more willing to participate and less shy in a separate group. Children without any obvious health problems were drawn from the general population through collaboration with local schools and sports clubs. To participate, the relevant local language had to be the main one spoken in the participant’s home.

The aim of the focus group interviews was to identify child-friendly labels normally used by the target group. In general, focus group interviews have predominantly been conducted with adults [15]. However, there is evidence that these are feasible with younger participants for use in the development of child-specific HRQoL instruments to gather information about the wording and vocabulary of children and adolescents themselves [15, 16].

Children and adolescents were first asked to talk about their own experiences with illness before being asked to describe pictures illustrating people with a health condition. This procedure aimed to identify words and phrases that children and adolescents used naturally and spontaneously when talking about health and illness. We were particularly interested in words young people used to describe the quantity or intensity of health problems. Subsequently, children were asked to rank the labels elicited from the earlier review of instruments between no problems to the most severe problems they could imagine. Lastly, the participants indicated the labels that they did not like or understand as well as those they liked the most.

##### Data analysis

The focus group discussions were documented by detailed notes or recorded and transcribed and then analyzed using thematic content analysis [17]. As typical for this kind of analysis, we defined categories (e.g., ‘labels mentioned by the participants themselves’, ‘information about the labels in the context of the ranking’) and screened the participants’ comments for statements referring to these categories. Based on the results from the review and the focus groups, each country identified a pool of potential labels for use with each dimension of the EQ-5D-Y.

#### Sorting and response scaling interviews

##### Procedure

Sorting and response scaling techniques were used in individual interviews with children and adolescents to determine the relative severity of each label identified in phase I. Sorting tasks were used with younger children (8–10 years) while older children (11–15 years) completed response scaling tasks.

A convenience sample of children and adolescents aged 8–15 years from the general population of school children recruited in primary and secondary schools was used. Different types of schools were included to ensure the participation of children and adolescents with different educational levels and socio-economic background. A total of 60 participants in each country was expected with 20 in each age group: 8–10 years, 11–2 years and 13–15 years. The sample size was somewhat larger than that used in developing the adult version EQ-5D-5L [7], as the surveyed population of children and adolescents was considered to be more heterogeneous in terms of age and verbal comprehension.

In the context of the development of HRQoL instruments, the response scaling method typically requires participants to assign a numeric rating to an item or label. This method has already been used in the development of other HRQoL instruments [18, 19]. In this study, older respondents (11–15 years) were asked to rate the severity of each label separately on a visual analog scale (VAS) from 0 to 10 (detailed labeling of the anchors see legend of Table 4). For younger children (8–10 years), the different categories of severity were presented on a ‘smiling face’ scale. Smiley faces are often used for child-friendly measures [20, 21]. We used a modified version of the faces scale from the UK Household Longitudinal Study [22]. For each label, the younger children were asked to choose one smiley out of five smileys (from 1 = smiley of very bad mood to 5 = smiley of very good mood). The anchors of the scale, so smiley 1 and smiley 5, were labelled in the same way as done for the anchors of the VAS that was used for the older participants.

All children rated all labels separately for each dimension. Participants were asked to indicate labels that they found hard to understand or which they did not use in daily language. Both the order in which dimensions and labels were presented to the participants were randomized to avoid bias. A pilot test of the tasks was conducted before being applied to the full sample.

##### Data analysis

Labels were first grouped into two categories (‘unusual and unclear labels’; ‘usual and easily understood labels’) based on participants’ comments. Mean (standard deviation), median, mode, minimum and maximum of the sorting and response scaling data were then computed for all labels using SPSS version 23. These analyses were done separately for younger and older participants as the scale for the younger participants ranged from 1 to 5 while it was 0 to 10 for the older participants.

##### Criteria for label selection

Labels were selected for further testing based primarily on their distribution along the severity continuum. As both 4L and 5L formats were being considered, two sets of criteria were specified as shown in Table 1.

**Table 1:** Definition of approximated mean values of the labels used for the two extended versions of the EQ-5D-Y

Version	Age group (years)	Development of targeted distances between labels	Approximated mean values and distances
4L	8–10	5 smilies; 4 labels needed	1.25–2.5–3.75–5
	11–15	VAS from 0 to 10; 4 labels needed	0–3.4–6.8–10
5L	8–10	5 smilies; 5 labels needed	1–2–3–4–5
	11–15	VAS from 0 to 10; 5 labels needed	0–2.5–5–7.5–10

Labels were considered appropriate for the extended versions, if the following criteria were met (ordered from most to least importance): (1) median and mode showed exactly the previously defined values (Table 1) or they were close to it, (2) median and mode had the same value, (3) standard deviation was very small, as that would show similarity of interpretation among respondents. The labels for the upper (‘unable to’, ‘extreme pain or discomfort’, ‘extremely worried, sad or unhappy’) and lower (‘no problems’, ‘no pain or discomfort’, ‘not worried, sad or unhappy’) levels of severity were selected as used for the anchors in the sorting and response scaling tasks as these showed good comprehensibility and feasibility.

If there were uncertainties about the final decision for a label and more than one label was appropriate for a severity level based on the quantitative results, the results of the qualitative data analysis were taken into account when making the final decision. At the end of this phase, draft 4L and 5L versions were available.

### Phase 2

#### Cognitive interviews

##### Procedure

Cognitive interviews were conducted to test the 4L and 5L draft versions for comprehensibility, feasibility and preferences between versions. In Germany, Spain and Sweden, healthy children and adolescents aged 8–15 years as well as those in treatment for a health condition participated in individual or group interviews. Healthy participants were recruited in collaboration with schools and participants with a health condition in collaboration with local hospitals. The interviews took place in a separate room assigned by the schools or hospitals. Participants with a health condition were included to get feedback from children who might use labels representing higher levels of severity.

According to the standardized protocol, participants first completed either the 4L or 5L to record their own health status, followed by a general discussion of the version. Participants then completed socio-demographic questions, before completing the other draft version and discussing that. Finally, they were asked which version they preferred and why. To avoid an ordering bias, the order of versions was varied. When discussing the versions, the paraphrasing method was used, whereby participants were asked to rephrase the items in their own words; probing was used to explore problems in answering, comprehension, and participants’ reasons for choosing a given response option [23].

In the UK, a slightly different approach was taken. Two focus group interviews were conducted to test the provisional 5L version. Pupils from primary and secondary schools participated. Recruitment of children with current experience of illness would have necessitated obtaining separate ethical approval from the National Health Service (NHS). This would have incurred significant delay so that recruitment was limited to children attending schools. Participants were initially asked to record their current health status using the 5L version and then to review the first page and to circle any words or phrases that might be difficult to understand for other people of their age. These words were discussed in the group. In addition, the participants reported how hard or easy they found it to answer the version. Finally, each pupil was asked to complete a written task designed to test their comprehension of key words and phrases that were considered to be the most problematic.

##### Data analysis

The interviews and group discussions were recorded, transcribed and analyzed using thematic content analysis [17]. Comments made by participants were assigned to defined categories such as ‘general comprehensibility and ease of use’, ‘comprehensibility of labels’, and ‘suggestions for changes’.

### Harmonization

As we wanted to develop language-specific versions from scratch, we did not expect to find absolute equivalent labels in all countries. However, the three 5L language versions (Swedish, Spanish, German) were translated into English and compared to each other and to the UK English version. Any discrepancies between versions were discussed in a harmonization exercise involving researchers from each country.

## Results

### Phase 1

#### Identifying a pool of possible labels for the severity levels

The review of HRQoL instruments and focus groups identified potentially usable labels (Germany: 79; Spain: 67; Sweden: 50; UK: 37) from which a smaller number of labels per dimension was selected for inclusion in the sorting and response scaling interviews (Table 2). During the screening of HRQoL instruments to select some candidate labels, the UK team was quite strict with regard to whether the labels were grammatically well compatible with the general format of EQ-5D dimension statements and whether they seemed to be child-friendly. This led to a smaller initial label pool than in other countries. The German label pool was quite big due to a complicated language that offered several options of wording. The German team wanted to give the chance to children and adolescents to give their view on many different labels. Labels representing the full range of severity were included in all countries. The same set of labels was applied for the ‘mobility’, ‘looking after myself’ and ‘usual activities’ dimensions and a somewhat different set for the ‘having pain or discomfort’ and ‘feeling worried, sad or unhappy’ dimensions.

**Table 2:** Number of severity labels selected for sorting and response scaling per dimension, by country

Dimension	Countries			
	Germany	Spain	Sweden	UK
Mobility	15	13	13	8
Looking after myself	15	13	13	8
Doing usual activities	15	13	13	8
Having pain or discomfort	16	11	12	7
Feeling worried sad or unhappy	12	12	11	7

#### Sorting and response scaling interviews

Each country conducted between 59 and 72 sorting and response scaling interviews giving a total of 255 interviews. Detailed information about the sample characteristics are shown in Table 3.

**Table 3:** Sample description for the sorting and response scaling interviews, by country

	Germany	Spain	Sweden	UK
*n*	64	72	60	58
	% (*n*)	% (*n*)	% (*n*)	% (*n*)
Gender				
Boys	43.8 (28)	50.0 (36)	38.3 (23)	46.6 (27)
Girls	56.3 (36)	50.0 (36)	61.7 (37)	53.4 (31)
Age-groups^a^(years)				
8–10	32.8 (21)	43.1 (31)	41.7 (25)	50.8 (30)
11–15	67.2 (43)	56.9 (41)	58.3 (35)	49.2 (28)
Chronic/long-lasting illness				
Yes	29.7 (19)	22.2 (16)	35.0 (21)	–
No	70.3 (45)	77.7 (56)	61.7 (39)	–
Don’t know	–	2.7 (2)	3.3 (2)	–
EQ-VAS (median)	90.0	95.0	86.0	88.0

^a^Dividing the age-groups like this is based on the split of age ranges in the field work in phase 1 and 2

Table 4 shows the range of median values for the ‘mobility’ dimension based on the responses of the participants aged 11–15 years, while Table 5 provides the same information for the ‘feeling worried, sad and unhappy’ dimension. These are provided as examples as the range of values for the other dimensions was similar. Labels covered an appropriate range of severity on the health continuum in all countries. The ratings from the participants aged 8–10 years were comparable to those from the older participants.

**Table 4:** Median VAS for the labels rated for the ‘mobility’ dimension in the response scaling interviews by participants aged 11–15 years, by country

Germany		Spain		Sweden		UK	
Labels ‘mobility’ dimension	Median value^a^	Labels ‘mobility’ dimension	Median value^b^	Labels ‘mobility’ dimension	Median value*	Labels ‘mobility’ dimension	Median value^c^
Keine	0	No	0	Inte	0	No	100
Kaum	0.9	Casi no	5	Pyttelite	1	A little bit	80
Ganz leichte	1	Escasos	20	Bara lite	2	A bit	70
Wenige	2	Un poco	30	Lite	2	Some	55
Leichte	2	Algunos	42	Lite grann	2	A lot	30
Ein wenig	2	Moderados	50	En aning	2.5	A great deal	20
Ein bisschen	2	Bastantes	65	Något	3	Terrible	10
Ein paar	3	Muchos	70	Ganska	6	Cannot	0
Einige	5	Muchisimos	75	Mycket	8	–	–
Viele	7.5	Severos	80	Väldigt	8	–	–
Große	8	Graves	85	Jätte	9	–	–
Sehr viele	8.5	Extremos	90	Extremt	9.5	–	–
Sehr große	9	No puedo	100	Kan inte	10	–	–
Extreme	9.8	–	–	–	–	–	–
Kann nicht	10	–	–	–	–	–	–

^a^VAS from 0 to 10 was used. Anchor ‘0’ was labelled as ‘no’ and anchor ‘10’ was labelled as ‘cannot’

^b^VAS from 0 to 100 was used. Anchor ‘0’ indicated the best status and anchor ‘100’ indicated the worst status

^c^VAS from 0 to 100 was used. Anchor ‘0’ was labelled as ‘cannot’ and anchor ‘100’ was labelled as ‘no’

**Table 5:** Median VAS for the labels rated for the ‘feeling worried, sad or unhappy’ dimension in the response scaling interviews by participants aged 11–15 years, by country

Germany		Spain		Sweden		UK	
Labels ‘feeling worried, sad or unhappy’ dimension	Median value^a^	Labels ‘feeling worried, sad or unhappy’ dimension	Median value^b^	Labels ‘feeling worried, sad or unhappy’ dimension	Median value^a^	Labels ‘feeling worried, sad or unhappy’ dimension	Median value^c^
Gar nicht	0	No tengo	0	Inte	0	Not	100
Nicht	0.2	Casi no tengo	5	Pyttelite	1	A little	85
Kaum	1.2	Un poco	15	Lite grann	1.9	A bit	70
Ein wenig	2	Algo	20	Lite	2	A lot	35
Ein bisschen	2	Una leve	35	Bara lite	2	Really	35
Leicht	2	Moderada	50	Något	3	Greatly	15
Etwas	3	Bastante	60	Ganska	5	Terribly	0
Große	8	Mucha	70	Mycket	8	–	–
Besonders	8	Severa	80	Väldigt	8	–	–
Sehr	8.5	Muchisimo	85	Jätte	9	–	–
Ganz	8.5	Extrema	90	Extremt	10	–	–
Total	9	Lo mas	100	–	–	–	–
Extrem	10	–	–	–	–	–	–

^a^VAS from 0 to 10 was used. Anchor ‘0’ was labelled as “not” and anchor ‘10’ was labelled as ‘extremely’

^b^VAS from 0 to 100 was used. Anchor ‘0’ indicated the best status and anchor ‘100’ indicated the worst status

^c^VAS from 0 to 100 was used. Anchor ‘0’ was labelled as ‘extremely’ and anchor ‘100’ was labelled as ‘not’

Median values, mode and standard deviation were considered in the decision regarding final labels for the 4L and 5L versions, as well as participants’ verbal statements. For example, the Swedish labels ‘pyttelite’ (a tiny bit) and ‘något’ (some, somewhat) for dimensions ‘mobility’, ‘looking after myself’ and ‘doing usual activities’ were ranked differently among the two age groups, who also appeared to interpret the words in different ways. Hence, these words were not chosen as final labels. The importance of the verbal statements was seen in Germany, where the label ‘ein bisschen’ (somewhat/a bit) was chosen for level 2 in the ‘feeling worried, sad or unhappy’ dimension. Based on the values given for the labels, ‘leicht’ (slightly) and ‘ein wenig’ (a few/a bit) were also possible options. However, participants mentioned that they would not use these words in everyday language in the context of being worried, sad or unhappy, so it was decided not to use them for this dimension.

### Phase 2

#### Cognitive interviews to test comprehension and feasibility of the extended 4L and 5L versions

Sample characteristics for participants in phase 2 are shown in Table 6. Participants’ comments indicated that they found both versions, EQ-5D-Y-4L and EQ-5D-Y-5L^1^, to be feasible to complete and easily understood*“…it wasn’t hard to complete and there were no difficult words”* [boy, 12 years, Sweden]. In Germany and Sweden, no questions were raised about the labels for the severity levels or the general questionnaires, among either the younger or older participants.

**Table 6:** Sample description for the participants of the cognitive interviews in phase 2, by country

	Germany	Spain	Sweden	UK
*n*	33	35	32	20
	% (*n*)	% (*n*)	% (*n*)	% (*n*)
Gender				
Boys	48.5 (16)	51.4 (18)	68.8 (22)	40.0 (8)
Girls	48.5 (16)	48.6 (17)	31.3 (10)	60.0 (12)
Missing	3.0 (1)	–	–	–
Age-groups^a^(years)				
8–10	33.3 (11)	42.9 (15)	65.6 (21)	50.0 (10)
11–15	66.6 (22)	57.1 (20)	34.4 (11)	50.0 (10)
Chronic/long-lasting illness				
Yes	51.5 (17)	54.3 (19)	46.9 (15)	–
No	48.5 (16)	45.7 (16)	49.9 (15)	–
Don’t know	0.0 (0)	0.0 (0)	6.3 (2)	–
EQ-VAS	Median	Median	Median	Mean
Participants from general population	85.0	96.0	95.5	90.0
Participants with a health condition	92.5*	89.0	85.0	–

*Two missing values

^a^Dividing the age-groups like this is based on the split of age ranges in the field work in phase 1 and 2

In Spain, some of the labels which were chosen after the sorting and response scaling exercises caused problems for the participants. For example, in the ‘mobility’, ‘looking after myself’, and ‘usual activities’ dimensions, level 2 ‘un poco de problema’ (a little bit of a problem) was changed to a more natural-sounding wording (‘algún pequeño problema’). Some of the younger participants were also unsure how to interpret ‘moderados’ and ‘moderadamente’ (moderate) and, after discussion between the researchers and children it was decided to use ‘bastante’ (quite a lot) as the alternative which was closest and easiest to understand. Other changes included replacing ‘muchísimos’ (used in the ‘mobility’, ‘looking after myself’, ‘doing usual activities’ and ‘pain and discomfort’ dimensions) with the more child-friendly terms ‘muchos’ or ‘mucho’ (a lot) and replacing ‘algo’ (somewhat) with ‘un poco’ (a little) in the ‘worried, sad or unhappy’ dimension.

When directly asked for their opinion, the majority of the participants in Germany, Spain and Sweden, irrespective of their health status, preferred the EQ-5D-Y-5L (Germany: 88%; Sweden: 66%; Spain: 68%) over the 4L version. They felt it allowed them to rate their health in more detail. They commented that the 5L version is more precise, and they liked the fact that it has a middle answer category. In Sweden, one respondent stated ‘*I thought the 5L version was best because there were more options to choose from’* [boy, 13 years, Sweden]. In Germany, one participant argued: ‘*[…] you are not able to state your current health status [in the 4L version] as precisely as in the 5L version’* [girl, 10 years, Germany]. Participants with health problems also noted that the EQ-5D-Y-5L provided more options for reporting severe health problems. Compared to the 5L version, some participants had the feeling that answering the 4L version was more difficult as there were fewer possibilities to choose from. However, two participants commented critically on the central response option in the 5L as they thought it might be used by respondents who were unwilling to decide between answers. However, the central response category of the EQ-5D-Y-3L has been used in previous studies without any evidence of this type of problem [6, 11, 12].

In the UK, only the EQ-5D-Y-5L was tested and no children reported difficulties in completing it. There were no questions from the participants while answering the questionnaire and no missing data. However, the discussion of the words ‘terrible’/‘terribly’ to describe level 5 in the dimensions ‘having pain or discomfort’ and ‘feeling worried, sad or unhappy’ with primary school children indicated the need for further attention as they were especially problematic. When language is embedded in a hierarchical structure then it could be assumed that the ‘correct’ understanding of a word is implied through its association with adjacent response categories. The label for severity level 5 defines the upper bound and has no scope for such a compensating mechanism. The UK team therefore considered the word ‘extreme’/‘extremely’ as a replacement for ‘terrible’/‘terribly’ since it was used in the other language versions. This was investigated in further interviews with a small number of children (*n* = 4) who confirmed this substitution.

The final language-specific 5L versions can be seen in Table 7, the 4L versions that were included in the testing can be found in the online resource 1 (Table A1).

**Table 7:** Final severity level labels of the EQ-5D-Y-5L

Language	Germany (German)	Spain (Spanish)	Sweden (Swedish)	UK (English)
Dimension	Bewegung (herumlaufen)	Moverse (Al caminar)	Kunna röra sig	Mobility (walking about)
Level 1	Ich habe keine Schwierigkeiten herumzulaufen	No tengo problemas para caminar	Jag har inte svårt att gå	I have no problems walking about
Level 2	Ich habe leichte Schwierigkeiten herumzulaufen	Tengo algún pequeño problema para caminar	Jag har lite svårt att gå	I have a little bit of a problem walking about
Level 3	Ich habe einige Schwierigkeiten herumzulaufen	Tengo bastantes problemas para caminar	Jag har ganska svårt att gå	I have some problems walking about
Level 4	Ich habe große Schwierigkeiten herumzulaufen	Tengo muchos problemas para caminar	Jag har väldigt svårt att gå	I have a lot of problems walking about
Level 5	Ich kann nicht herumlaufen	No puedo caminar	Jag kan inte gå	I cannot walk about

Dimension	Für mich selbst sorgen	Cuidar de mí mismo	Ta hand om mig själv	Looking after myself
Level 1	Ich habe keine Schwierigkeiten mich selber zu waschen oder anzuziehen	No tengo problemas para lavarme o vestirme sólo	Jag har inte svårt att tvätta mig eller klä på mig själv	I have no problems washing or dressing myself
Level 2	Ich habe leichte Schwierigkeiten mich selber zu waschen oder anzuziehen	Tengo algún pequeño problema para lavarme o vestirme sólo	Jag har lite svårt att tvätta mig eller klä på mig själv	I have a little bit of a problem washing or dressing myself
Level 3	Ich habe einige Schwierigkeiten mich selber zu waschen oder anzuziehen	Tengo bastantes problemas para lavarme o vestirme sólo	Jag har ganska svårt att tvätta mig eller klä på mig själv	I have some problems washing or dressing myself
Level 4	Ich habe große Schwierigkeiten mich selber zu waschen oder anzuziehen	Tengo muchos problemas para lavarme o vestirme sólo	Jag har väldigt svårt att tvätta mig eller klä på mig själv	I have a lot of problems washing or dressing myself
Level 5	Ich kann mich nicht selber waschen oder anziehen	No puedo lavarme o vestirme sólo	Jag kan inte tvätta mig eller klä på mig själv	I cannot wash or dress myself

Dimension	Was ich normalerweise tue (zum Beispiel: in die Schule gehen, Hobbys, Sport, Spielen, Dinge mit Familie und Freunden machen)	Hacer actividades habituales (Ej. Ir al colegio, al hacer deporte, al jugar, al hacer actividades con la familia o los amigos…)	Göra vanliga aktiviteter (till exempel gå i skolan, sport-och fritidsaktiviteter, lek, göra saker med familj eller kompisar)	Doing usual activities (for example, going to school, hobbies, sports, playing, doing things with family or friends)
Level 1	Ich habe keine Schwierigkeiten das zu tun, was ich normalerweise tue	No tengo problemas para hacer mis actividades habituales	Jag har inte svårt att göra mina vanliga aktiviteter	I have no problems doing my usual activities
Level 2	Ich habe leichte Schwierigkeiten das zu tun, was ich normalerweise tue	Tengo algún pequeño problema para hacer mis actividades habituales	Jag har lite svårt att göra mina vanliga aktiviteter	I have a little bit of a problem doing my usual activities
Level 3	Ich habe einige Schwierigkeiten das zu tun, was ich normalerweise tue	Tengo bastantes problemas para hacer mis actividades habituales	Jag har ganska svårt att göra mina vanliga aktiviteter	I have some problems doing my usual activities
Level 4	Ich habe große Schwierigkeiten das zu tun, was ich normalerweise tue	Tengo muchos problemas para hacer mis actividades habituales	Jag har väldigt svårt att göra mina vanliga aktiviteter	I have a lot of problems doing my usual activities
Level 5	Ich kann nicht das tun, was ich normalerweise tue	No puedo hacer mis actividades habituales	Jag kan inte göra mina vanliga aktiviteter	I cannot do my usual activities

Dimension	Schmerzen oder körperliche Beschwerden	Tener dolor o sentirse mal	Ha ont eller ha besvär	Having pain or discomfort
Level 1	Ich habe keine Schmerzen oder körperlichen Beschwerden	No tengo dolor ni me siento mal	Jag har inte ont eller inte några besvär	I have no pain or discomfort
Level 2	Ich habe leichte Schmerzen oder körperlichen Beschwerden	Tengo un poco de dolor o me siento un poco mal	Jag har lite ont eller lite besvär	I have a little bit of pain or discomfort
Level 3	Ich habe einige Schmerzen oder körperliche Beschwerden	Tengo bastante dolor o me siento bastante mal	Jag har ganska ont eller ganska mycket besvär	I have some pain or discomfort
Level 4	Ich habe große Schmerzen oder körperliche Beschwerden	Tengo mucho dolor o me siento muy mal	Jag har väldigt ont eller väldigt mycket besvär	I have a lot of pain or discomfort
Level 5	Ich habe extreme Schmerzen oder körperliche Beschwerden	Tengo dolor extremo o me siento extremadamente mal	Jag har extremt ont eller extremt mycket besvär	I have extreme pain or discomfort

Dimension	Sich unglücklich, traurig oder besorgt fühlen	Sentirse preocupado, triste o infeliz	Känna sig orolig, ledsen eller olycklig	Feeling worried, sad or unhappy
Level 1	Ich bin nicht unglücklich, traurig oder besorgt	No me siento preocupado, triste o infeliz	Jag är inte orolig, ledsen eller olycklig	I am not worried, sad or unhappy
Level 2	Ich bin ein bisschen unglücklich, traurig oder besorgt	Me siento un poco preocupado, triste o infeliz	Jag är lite orolig, ledsen eller olycklig	I am a little bit worried, sad or unhappy
Level 3	Ich bin etwas unglücklich, traurig oder besorgt	Me siento bastante preocupado, triste o infeliz	Jag är ganska orolig, ledsen eller olycklig	I am quite worried, sad or unhappy
Level 4	Ich bin sehr unglücklich, traurig oder besorgt	Me siento muy preocupado, triste o infeliz	Jag är väldigt orolig, ledsen eller olycklig	I am really worried, sad or unhappy
Level 5	Ich bin extrem unglücklich, traurig oder besorgt	Me siento extremadamente preocupado, triste o infeliz	Jag är extremt orolig, ledsen eller olycklig	I am extremely worried, sad or unhappy

© 2018 EuroQol Group. EQ-5D™ is a trade mark of the EuroQol Group

### Harmonization

The comparison of the 5L versions occasionally showed divergent wordings for the labels. This was primarily due to (1) difficulties finding an exact translation for a term in English or (2) because a specific label was chosen based on participants’ comments and therefore justified by the results of the field work. For example, the fourth level of the first three dimensions is ‘a lot of’ in English and—more or less—also in Swedish but ‘große’ (great) in German. The two labels are therefore not strictly equivalent but the alternative German wording of ‘viele’ (a lot of) was more frequently cited by participants in the German cognitive debriefing exercise as being unclear and an unusual wording. The term ‘große’ was therefore preferred. This also means that the youth version is consistent with the wording used in the German 5L adult version. The discussion of all labels and the slight discrepancies in the different languages showed that the labels were comparable, i.e., labels remained as developed by the national teams.

## Discussion

In a process of identifying appropriate labels for an extended version of the EQ-5D-Y and testing the comprehensibility and feasibility, this study was successful in establishing a 5L version of the EQ-5D-Y, the EQ-5D-Y-5L.

It is hoped that the development of this 5L version will lead to an improvement over the EQ-5D-Y-3L in terms of its performance in general and sensitivity in particular. Compared to the EQ-5D-Y-3L, which defines 243 health states, the EQ-5D-Y-5L defines a broader spectrum of 3125 possible health states. However, the 5L version will require further investigation in terms of testing its psychometric properties. The 5L format maintains comparability with the corresponding adult version as do the adult and youth version of EQ-5D-3L. It is anticipated that this will allow continuous measurement of health status over a lifetime and also to permit comparison of results obtained using the two versions of the instrument [11]. This can be important when evaluating the impact of chronic disease which appears in childhood and lasts throughout adulthood [24].

As recommended in guidelines on the development of patient reported outcome (PRO) instruments, we made considerable efforts to take into account the views of the target group when developing the instrument [25–27]. Standardized procedures were co-designed across national research teams with children and adolescents being involved in the process at several stages to ensure development of an age appropriate instrument by using their preferred wording and everyday language wherever possible. In phase 1, participants reviewed preexisting labels as well as suggested possible new labels for use in the new version of the questionnaire based on understandable language and everyday speech of children. We found that participants of all ages were able to rate the severity of different labels using a sorting or response scaling task. This study is to the best of our knowledge the first to demonstrate the feasibility of using response scaling tasks in participants as young as eight. Participants contributed actively in phase 2 interviews and freely expressed their opinions about the different wording options offered. Overall, the ability of young persons to participate in studies using scientific methods should not be underestimated. The recruitment of children and adolescents as study participants is a challenge; in this study, it was especially difficult to recruit those with a health condition. Ideally, for the integration of the young peoples’ perspective, it is necessary to involve them directly in research. Overall, the applied methods worked well in all countries, although the protocol adopted in the UK and in Spain deviated marginally from that employed elsewhere.

Comparing the EQ-5D-Y-5L and EQ-5D-Y-3L labels shows that the structure was not always changed in a similar way in all countries. Some of the labels from EQ-5D-Y-3L remained, while others were replaced. As it was found in the development of the adult EQ-5D-5L [28, 29], it would have been overly simplistic to simply insert an additional level between the original levels 1 and 2 and levels 2 and 3. Hence, it was important to examine different labels for use in the extended versions.

This work on severity labels of the EQ-5D-Y descriptive system is also important in the context of future development of national value sets where the labels have to be valued as part of health state profiles, i.e., without the respondent seeing all severity labels of one dimension and their complete rank order as in the whole descriptive system.

A limitation of our study is that we used convenience samples in all countries and within both study phases; hence, the study population is not representative of the national population in each country. However, by including all age groups, boys and girls, and participants from different types of schools, we tried to ensure the inclusion of children and adolescents with a broad spread of characteristics.

The present study has produced a self-report version of the EQ-5D-Y-5L but future research will be needed to develop proxy-versions of the instrument. In the future, it will be important to conduct validation studies for the different language versions of EQ-5D-Y-5L in different groups of children and adolescents and especially among participants with different health conditions, to identify measurement properties of the instrument. The UK English version  is assumed to be the source version for the translation of further language versions. It is also expected that research on valuation of the EQ-5D-Y-3L and EQ-5D-Y-5L will go on.

## Conclusion

Children and adolescents in all participating countries contributed to the selection of candidate labels for an enhanced version of the EQ-5D-Y-3L and were able to rate the severity of different labels. They preferred the five-level version of EQ-5D-Y over the proposed four-level alternative. The new EQ-5D-Y-5L was comprehensible and feasible for children and adolescents in the age range 8–15 years and should provide a useful tool for those wishing to incorporate a short, simple, and easy to use measure of health status in their research. Before being used more extensively, further research to test the psychometric performance of the EQ-5D-Y-5L is required as well as an investigation of its feasibility for use in health state valuation exercises.

## Electronic supplementary material

Below is the link to the electronic supplementary material.

Supplementary material 1 (DOCX 34 KB)

## Footnotes

1 In the UK, only a 5L version was developed and tested.
