# Supplementary material for: EQ-5D-Y-5L: developing a revised EQ-5D-Y with increased response categories
Source: Qual Life Res. 2019 Feb 9;28(7):1951–61. doi: 10.1007/s11136-019-02115-x (PMC6571085; doi:10.1007/s11136-019-02115-x)
Supplement: Supplementary file 1 — Supplementary material 1 (DOCX 34 KB) [file 11136_2019_2115_MOESM1_ESM.docx]

**Title Page**

**Title:**

EQ-5D-Y-5L - Developing a revised EQ-5D-Y with increased response categories

**Names of authors:**

Simone Kreimeier^1^

Mimmi Åström^2,3,4^

Kristina Burström^2,3,4^

Ann-Charlotte Egmar^2,5^

Narcis Gusi^6^

Michael Herdman^7^

Paul Kind^8, 9^

Miguel A. Perez-Sousa^6^

Wolfgang Greiner^1^

***Electronic appendix***

**Table A1: Final labels for the extended 4L version of the EQ-5D-Y**

|  | **Germany (German)** | **Sweden (Swedish)** | **Spain (Spanish)** |
| --- | --- | --- | --- |
| **Mobility** *(walking about)* | | | |
| **Dimension** | **Bewegung** *(herumlaufen)* | **Kunna röra sig** | **Moverse** *(Al caminar)* |
| **Level 1** | Ich habe keine Schwierigkeiten herumzulaufen | Jag har inte svårt att gå | No tengo problemas para caminar |
| **Level 2** | Ich habe ein paar Schwierigkeiten herumzulaufen | Jag har lite svårt att gå | Tengo un poco de problema para caminar |
| **Level 3** | Ich habe viele Schwierigkeiten herumzulaufen | Jag har väldigt svårt att gå | Tengo bestantes problemas para caminar |
| **Level 4** | Ich kann nicht herumlaufen | Jag kan inte gå | Tengo muchísimos problemas para caminar |
| **Looking after myself** | | | |
| **Dimension** | **Für mich selbst sorgen** | **Ta hand om mig själv** | **Cuidar de mí mismo** |
| **Level 1** | Ich habe keine Schwierigkeiten mich selber zu waschen oder anzuziehen | Jag har inte svårt att tvätta mig eller klä på mig själv | No tengo problemas para lavarme o vestirme sólo |
| **Level 2** | Ich habe ein paar Schwierigkeiten mich selber zu waschen oder anzuziehen | Jag har lite svårt att tvätta mig eller klä på mig själv | Tengo un poco de problema para lavarme o vestirme sólo |
| **Level 3** | Ich habe viele Schwierigkeiten mich selber zu waschen oder anzuziehen | Jag har väldigt svårt att tvätta mig eller klä på mig själv | Tengo bestantes problemas para lavarme o vestirme sólo |
| **Level 4** | Ich kann mich nicht selber waschen oder anziehen | Jag kan inte tvätta mig eller klä på mig själv | Tengo muchísimos problemas para lavarme o vestirme sólo |
| **Doing usual activities** *(for example, going to school, hobbies, sports, playing, doing things with family or friends)* | | | |
| **Dimension** | **Was ich normalerweise tue** *(zum Beispiel: in die Schule gehen, Hobbys, Sport, Spielen, Dinge mit Familie und Freunden machen)* | **Göra vanliga aktiviteter** *(till exempel gå i skolan, sport-och fritidsaktiviteter, lek, göra saker med familj eller kompisar)* | **Hacer actividades habituales** *(Ej. Ir al colegio, al hacer deporte, al jugar, al hacer actividades con la familia o los amigos…)* |
| **Level 1** | Ich habe keine Schwierigkeiten das zu tun, was ich normalerweise tue | Jag har inte svårt att göra mina vanliga aktiviteter | No tengo problemas para hacer mis actividades habituales |
| **Level 2** | Ich habe ein paar Schwierigkeiten das zu tun, was ich normalerweise tue | Jag har lite svårt att göra mina vanliga aktiviteter | Tengo un poco de problema para hacer mis actividades habituales |
| **Level 3** | Ich habe viele Schwierigkeiten das zu tun, was ich normalerweise tue | Jag har väldigt svårt att göra mina vanliga aktiviteter | Tengo bestantes problemas para hacer mis actividades habituales |
| **Level 4** | Ich kann nicht das tun, was ich normalerweise tue | Jag kan inte göra mina vanliga aktiviteter | Tengo muchísimos problemas para hacer mis actividades habituales |
| **Having pain or discomfort** | | | |
| **Dimension** | **Schmerzen oder  körperliche Beschwerden** | **Ha ont eller ha besvär** | **Tener dolor o sentirse mal** |
| **Level 1** | Ich habe keine Schmerzen oder körperlichen Beschwerden | Jag har inte ont eller inte några besvär | No tengo dolor ni me siento mal |
| **Level 2** | Ich habe ein paar Schmerzen oder körperlichen Beschwerden | Jag har lite ont eller lite besvär | Tengo algo de dolor o me siento mal |
| **Level 3** | Ich habe viele Schmerzen oder körperliche Beschwerden | Jag har väldigt ont eller väldigt mycket besvär | Tengo un dolor o malestar moderado |
| **Level 4** | Ich habe extreme Schmerzen oder körperliche Beschwerden | Jag har extremt ont eller extremt mycket besvär | Tengo muchísimo dolor o me siento muy mal |
| **Feeling worried, sad or unhappy** | | | |
| **Dimension** | **Sich unglücklich, traurig oder  besorgt fühlen** | **Känna sig orolig, ledsen eller olycklig** | **Sentirse preocupado, triste o infeliz** |
| **Level 1** | Ich bin nicht unglücklich, traurig oder besorgt | Jag är inte orolig, ledsen eller olycklig | No me siento preocupado, triste o infeliz |
| **Level 2** | Ich bin etwas unglücklich, traurig oder besorgt | Jag är lite orolig, ledsen eller olycklig | Me siento algo preocupado, triste o infeliz |
| **Level 3** | Ich bin sehr unglücklich, traurig oder besorgt | Jag är väldigt orolig, ledsen eller olycklig | Me siento moderadamente preocupado, triste o infeliz |
| **Level 4** | Ich bin extrem unglücklich, traurig oder besorgt | Jag är extremt orolig, ledsen eller olycklig | Me siento muy preocupado, triste o infeliz |
